# MicroRNA29B induces fetal hemoglobin *via* inhibition of the HBG repressor protein MYB *in vitro* and in humanized sickle cell mice

**DOI:** 10.3389/fmed.2022.1043686

**Published:** 2022-11-25

**Authors:** Qingqing Gu, Chithra D. Palani, Alana Smith, Biaori Li, Ernestine Kubi Amos-Abanyie, Ugochi Ogu, Lu Lu, Betty S. Pace, Athena Starlard-Davenport

**Affiliations:** ^1^Department of Genetics, Genomics, and Informatics, College of Medicine, The University of Tennessee Health Science Center, Memphis, TN, United States; ^2^Department of Pediatrics, Division of Hematology/Oncology, Augusta University, Augusta, GA, United States; ^3^Department of Biochemistry and Molecular Biology, Augusta University, Augusta, GA, United States; ^4^Center for Sickle Cell Disease, Department of Medicine-Hematology, The University of Tennessee Health Science Center, Memphis, TN, United States; ^5^Center for Sickle Cell Disease, The University of Tennessee Health Science Center, Memphis, TN, United States

**Keywords:** MIR29B, fetal hemoglobin (HbF), MYB, HBG, sickle cell

## Abstract

**Introduction:**

Therapeutic strategies aimed at reactivating *HBG* gene transcription and fetal hemoglobin (HbF) synthesis remain the most effective strategy to ameliorate the clinical symptoms of sickle cell disease (SCD). We previously identified microRNA29B (MIR29B) as a novel HbF inducer *via* targeting enzymes involved in DNA methylation. We provided further evidence that the introduction of MIR29B into KU812 leukemia cells significantly reduced MYB protein expression. Therefore, the aim of this study was to determine the extent to which MIR29B mediates HbF induction *via* targeting *MYB* in KU812 leukemia cells and human primary erythroid progenitors and to investigate the role of MIR29B in HbF induction *in vivo* in the humanized Townes SCD mouse model.

**Materials and methods:**

Human KU812 were cultured and normal CD34 cells (*n* = 3) were differentiated using a two-phase erythropoiesis culturing system and transfected with MIR29B (50 and 100 nM) mimic or Scrambled (Scr) control *in vitro*. A luciferase reporter plasmid overexpressing MYB was transfected into KU812 cells. Luciferase activity was quantified after 48 h. Gene expression was determined by quantitative real-time PCR. *In vivo* studies were conducted using Townes SCD mice (6 per group) treated with MIR29B (2, 3, and 4 mg/kg/day) or Scr control by 28-day continuous infusion using subcutaneous mini osmotic pumps. Blood samples were collected and processed for complete blood count (CBC) with differential and reticulocytes at weeks 0, 2, and 4. Flow cytometry was used to measure the percentage of HbF-positive cells.

**Results:**

*In silico* analysis predicted complementary base-pairing between MIR29B and the 3′-untranslated region (UTR) of *MYB*. Overexpression of MIR29B significantly reduced *MYB* mRNA and protein expression in KU812 cells and erythroid progenitors. Using a luciferase reporter vector that contained the full-length *MYB* 3′-UTR, we observed a significant reduction in luciferase activity among KU812 cells that co-expressed MIR29B and the full-length *MYB* 3′-UTR as compared to cells that only expressed *MYB* 3′-UTR. We confirmed the inhibitory effect of a plasmid engineered to overexpress *MYB* on *HBG* activation and HbF induction in both KU812 cells and human primary erythroid progenitors. Co-expression of MIR29B and *MYB* in both cell types further demonstrated the inhibitory effect of MIR29B on *MYB* expression, resulting in *HBG* reactivation by real-time PCR, Western blot, and flow cytometry analysis. Finally, we confirmed the ability of MIR29B to reduce sickling and induce HbF by decreasing expression of *MYB* and *DNMT3* gene expression in the humanized Townes sickle cell mouse model.

**Discussion:**

Our findings support the ability of MIR29B to induce HbF *in vivo* in Townes sickle cell mice. This is the first study to provide evidence of the ability of MIR29B to modulate *HBG* transcription by *MYB* gene silencing *in vivo*. Our research highlights a novel MIR-based epigenetic approach to induce HbF supporting the discovery of new drugs to expand treatment options for SCD.

## Introduction

Sickle cell disease (SCD) is a common genetic red blood cell disorder that affects over 20 million individuals worldwide (1). People with SCD have abnormal hemoglobin S (HbS) molecules, resulting from an A to T mutation in codon 6 of the *HBB* gene (2, 3). Under low oxygen conditions, HbS molecules polymerize and erythrocytes sickle, which can result in vaso-occlusion. Therapeutic strategies aimed at reactivating *HBG* gene transcription and fetal hemoglobin (HbF) synthesis remains the most effective strategy to ameliorate the clinical symptoms of SCD, including vaso-occlusive crises (4, 5).

Recently microRNAs (miRNAs) have emerged as a novel class of potential therapeutics due to their ability to restore expression of genes involved in tumor suppression (6), aging (7), and various human diseases (8–11). MiRNAs are endogenous, small (∼22 nt in size) regulatory RNA molecules that function to modulate post-transcriptional gene silencing through complimentary base-pair binding to their target mRNAs (12). MiRNAs are naturally expressed at varying levels in mammalian tissues, including blood plasma, and serum (13). Studies also provide evidence for a role of miRNAs in directly targeting *HBG* gene expression or transcriptional repressors of *HBG* gene expression during hemoglobin switching (14, 15). Thus, miRNAs that target genes involved in regulating *HBG* gene expression may serve as attractive therapeutic candidates for HbF induction.

Previously, we demonstrated that microRNA29B (MIR29B) reactivated *HBG* gene transcription and induced HbF expression *in vitro* by inhibiting the *de novo* DNA methyltransferases, *DNMT3A* and *DNMT3B* (16). Our findings further suggested that MIR29B may also target the *HBG* transcriptional repressor protein MYB in KU812 leukemia cells (16). Here, we have expanded our findings and show for the first time that MIR29B reactivates *HBG* gene transcription and induces HbF expression by silencing the *HBG* repressor protein MYB *in vitro* in KU812 leukemia cells and normal human erythroid progenitors generated from CD34^+^ stem cells and in the preclinical Townes SCD mouse model. Our preclinical findings highlight the therapeutic potential of MIR29B as a promising treatment for inducing HbF in SCD and other β-hemoglobinopathies.

## Materials and methods

### *In vitro* cell culture with microRNA29B and co-transfection with MYB DNA

Human KU812 leukemia cells were cultured in Iscove’s Modified Dulbecco Medium with 10% fetal bovine serum as previously published by our lab (16). Cells were harvested for cell count and viability using 0.4% Trypan blue exclusion assay. Cells were seeded at a density of 0.5 × 10^6^ viable cells per 100 mm plate for different treatments. During log phase growth, KU812 cells were transfected with 50 and 100 nmol/l of pre-MIR29B (Applied Biosystems, Waltham, MA, USA) or Scrambled (Scr) oligonucleotide control (100 nmol/l) for 48 h in three independent replicates using Opti-MEM media (Gibco, Waltham, MA, USA) and Lipofectamine™ 2000 transfection reagent (Invitrogen Carlsbad, CA, USA) according to the manufacturer’s instructions, then harvested for subsequent analyses. KU812 cells were also co-transfected with pGL3-*MYB*-3′-untranslated region (UTR) (Addgene plasmid # 25,798, Watertown, MA, USA), which contains the full length *MYB* 3′UTR cloned into the *Xba*I/*Sal*I restriction sites of the pGL3-control luciferase reporter vector (17). The human tagged ORF clone engineered to overexpress c-*MYB* (Origene, Rockville, MD, USA) was used alone or co-transfected with MIR29B.

### Erythroid differentiation of human CD34^+^ stem cells and microRNA29B co-electroporation with MYB DNA

Human bone marrow CD34^+^ stem cells (ReachBio, Seattle, WA, USA) were cultured in a modified two-phase liquid culture system as previously published (18). During phase I, stem cells were grown in minimum essential medium-α (αMEM) containing AB serum, interleukin-3 (10 ng/ml), stem cell factor (10 ng/ml), and erythropoietin (2 IU/ml). On day 7, cells transitioned to Phase II media where they remained under erythropoietin (2 IU/ml) stimulation. Erythroid progenitors were electroporated on day 8 with human mature MIR29B or Scr mimic or with c-*MYB* overexpression plasmid using the Amaxa^®^ Human CD34^+^ Cell Nucleofector^®^ Kit. After 48 h, cells were harvested for reverse transcription-quantitative PCR (RT-qPCR), Western blot, and flow cytometry analysis. Giemsa staining was used to monitor cell morphology and cell counts; viability was monitored using 0.4% Trypan blue exclusion assay (Gibco, Carlsbad CA, USA).

### RNA isolation and RT-qPCR analysis

Total RNA was isolated as previously published (16). To quantify mRNA levels for *MYB*, *HBG*, and *HBB* and the internal control β-actin, gene specific primers were used (Supplementary Table 1). All mRNA levels were normalized to β-actin before analysis. Quantification of MIR29B was performed using the TaqMan miRNA assay (Applied Biosystems, Waltham, MA, USA) according to the manufacturer’s instructions and *RNU48* was used as endogenous control. The 2^–ΔΔCt^ method was used for calculating the relative amount of target mRNA. All RT-qPCR reactions were performed in triplicate, repeated at least three times, and always included a no-template sample as a negative control. RT-qPCR results are presented as average fold change of target gene in cells relative to Scr control, which was normalized to one.

### Western blot analysis

Total protein was isolated and Western blot analysis was performed as previously published (16). Primary antibodies against MYB (59995S), HbF (39386S), and HbA (84934S) were purchased from Cell Signaling Technology (Danvers, MA, USA) and diluted in the range of 1:250 to 1:2000, incubated overnight and then followed by treatment with secondary antibody. The primary antibody against β-actin (AM4302), the internal control, was purchased from Invitrogen (Waltham, MA, USA).

#### Animal models and drug treatment

The humanized Townes SCD transgenic mouse (B6; 129-Hbatm1 [HBA] Tow Hbbtm2 [HBG1, HBB*] Tow/Hbbtm3 [HBG1, HBB] Tow/J), which completes hemoglobin switching from human γ-globin to β^s^-globin shortly after birth (19), were purchased from Jackson Laboratories. Mice were maintained and genotyping was performed with gene specific primers. All animal studies were approved by the Augusta University Institutional Animal Care and Use Committee. Townes SCD mice ages 4–6 months old, 4–10 mice per group (equal males and females), were treated by 28 days of continuous infusion using surgically implanted subcutaneous Alzet mini-osmotic pumps (DURECT corporation, Alzet Osmotic Pumps, Cupertino, CA, USA). Drug treatment groups in SCD mice included: (1) 2 mg/kg/day Scr control, (2) 2 mg/kg/day MIR29B mimic, (3) 3 mg/kg/day Scr control, (4) 3 mg/kg/day MIR29B mimic, (5) 4 mg/kg/day Scr control, and (6) 4 mg/kg/day MIR29B mimic. MIR29B mimics were purchased from Dharmacon (Lafayette, CO, USA). Mice were weighed at week 0 before and after pump placement and then again at weeks 2 and 4. Blood samples were collected in BD Microtainer Capillary blood collection K2-EDTA tubes at weeks 0, 2, and 4 and were processed for complete blood count (CBC) with differential. The percentage of reticulocytes (acridine orange) was determined by flow cytometry. To quantify MIR29B levels, total RNA was isolated using TRIzol and RT-qPCR analysis was performed.

#### Alzet mini-osmotic pump implantation

Before surgery, Buprenorphine SR was given for pain and then mice anesthetize with Isoflurane by inhalation. Once the animal was anesthetized, the area where pump was placed was shaved and then cleaned with a surgical scrub consisting of alternating betadine and alcohol wipes. Lidocaine was injected locally followed by a half-inch mid-scapular incision for pump placement on the back of the animal. A hemostat was used to spread the incision and subcutaneous tissue to create a pocket for the pump. Scr control or MIR29B filled Alzet mini-osmotic pump was inserted with the delivery portal first and wound closed with wound clips. Mice were monitored daily and pain was addressed with an additional dose of Buprenorphine SR. Once the mice wound was healed, clips were removed, and the blood samples were collected at weeks 0, 2, and 4 for analysis.

#### Complete blood count with differential

Peripheral blood from 4–6 months old mice was collected in BD Microtainer Lithium heparin tubes by tail vein bleeding. Automated CBC and differentials were completed on the Micros 60 CS/CT machine (HORIBA Medical/ABX Diagnostics, Irvine, CA, USA) according to the manufacturer’s protocol.

#### *In vitro* RBC sickling analysis

In a BD Vacutainer EDTA tube, peripheral blood was collected through tail bleeding and washed with 1X phosphate buffered saline. Cells were suspended 1:300 ratio in Iscove’s Modified Dulbecco’s Medium containing 10% heat inactivated fetal bovine serum, and incubated at 37°C in normoxic (21%) and hypoxic (1%) conditions for 12 h in a O_2_ hypoxic chamber (Coy Laboratory Products, Grass Lake, MI, USA). Subsequently, blood samples were treated with 4% formaldehyde for 10 min and then transferred to room temperature. Using light microscopy, sickling of red blood cells were quantified by changes in cell morphology. 20X magnification of bright field images were attained on an EVOS Cell Imaging systems (Thermo Fisher Scientifics, Waltham, MA, USA).

#### Flow cytometry analysis

To measure the percentage of HbF positive cells (F-cells), mouse peripheral blood samples were washed using cold PBS containing 0.5% BSA and fixed with 4% paraformaldehyde. Centrifuged to discard the supernatant, cells were washed and permeabilized with 1:1 of Acetone and Methanol and then stained with FITC conjugated sheep anti-human HbF antibody (Cat. #A80-136) purchased from Bethyl Laboratories, Inc. (Montgomery, TX, USA). Sheep IgG Isotype control was used to detect non-specific staining. The cells were washed once with phosphate buffered saline containing 0.5% bovine serum albumin buffer, and F-cells were measured and quantified by flow cytometry (20) using AttuneTM NxT Flow Cytometer and Attune and Novex software (ThermoFisher Scientific, Rockland, IL, USA).

### Statistical analysis

Data from at least 3–6 replicates of independent experiments were reported as the mean ± standard error of the mean (SEM). Data was analyzed by ANOVA or by a two-tailed Student’s *t*-test to determine statistical significance. Statistical analysis was completed by unpaired student’s *t*-test and ANOVA for *N* = 6 mice per group for two independent experiments. *P* < 0.05 was considered statistically significant.

## Results

**FIGURE 1:**
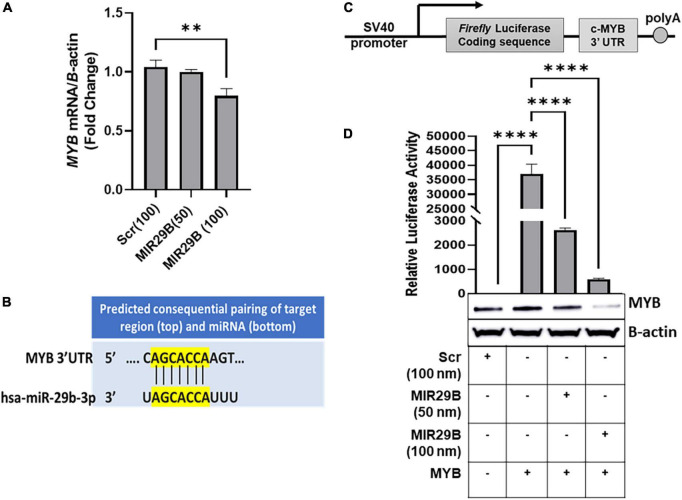
MicroRNA29B (MIR29B) targets 3′-untranslated region (UTR) of *MYB* in KU812 cells. **(A)** Real-time PCR analysis of *MYB* mRNA relative to β-actin internal control in KU812 cells transfected with MIR29B mimic (50 and 100 nM) or scrambled (Scr) control (100 nm). **(B)** Complementary sequence for MIR29B and the 3 – UTR of *MYB.* **(C)** Luciferase reporter construct containing the c-*MYB* 3′-UTR. **(D)** Luciferase activity in KU812 cells 48 h after transfection with MIR29B mimic (50 and 100 nM) and *MYB* 3′-UTR reporter plasmid. Accompanied Western blot showing expression of MYB protein in cells co-transfected with MYB overexpression plasmid and MIR29B mimic or Scr control (100 nm). The + and – signs indicate the presence or absence of MYB or MIR29B overexpression in KU812 cells. Data are shown at the mean ± standard error of the mean (SEM). **P < 0.005, ***P < 0.0005, and ****P < 0.0001 is statistically significant.

We previously showed that overexpression of MIR29B resulted in a significant decrease in expression of MYB, a known *HBG* repressor protein, in KU812 leukemia cells (16). To determine the effect of exogenous MIR29B expression on *MYB* gene silencing, we initially introduced MIR29B mimic (50 and 100 nM) into KU812 cells, which expresses the *HBG* and *HBB* genes, and measured *MYB* mRNA expression using RT-qPCR (Figure 1). We observed that transfection of MIR29B mimic (100 nM) for 48 h significantly decreased *MYB* mRNA expression by 25% as compared to Scr control (100 nM) cells (*p* < 0.01) (Figure 1A). Therefore, we utilized KU812 cells to determine whether MIR29B alters *MYB* expression as a mechanism of HbF induction.

Because miRNAs are known to post-transcriptionally regulate expression of their target mRNAs *via* binding to the 3′-UTR to repress protein production, we hypothesized that MIR29B might target the 3′-UTR of *MYB*. Using *in silico* analysis, we discovered that MIR29B has a consensus sequence complimentary to the 3′-UTR of *MYB* (Figure 1B). Thus, we wanted to determine whether *MYB* is regulated by MIR29B in KU812 cells. We transfected a luciferase reporter plasmid containing the full-length *MYB* 3′-UTR sequence. The sequence replaces the SV40 enhancer and SV40 poly (A) signal of pGL3-control (17) (Figure 1C) into KU812 cells alone or in combination with MIR29B mimic (50 and 100 nM) or Scr control (100 nM) and assessed luciferase activity (Figure 1D). In cells that overexpressed the full length *MYB* 3′-UTR alone, luciferase activity was significantly increased (*P* < 0.0001) as compared to Scr control cells (Figure 1D). However, when exogenous MIR29B mimic (50 and 100 nM) was co-expressed with the full length *MYB* 3′-UTR, luciferase activity was significantly reduced by up to 16-fold (MIR29B 50 nM, *p* < 0.001) and 80-fold (MIR29B 100 nM, *p* < 0.001) as compared to KU812 cells transfected with the full length *MYB* 3′-UTR alone (Figure 1D). Western blot analysis further confirmed the inhibitory effect of introducing MIR29B (100 nM) on MYB protein expression (Figure 1D). Specifically, co-transfection of MIR29B mimic (100 nM) and a plasmid engineered to overexpress c-*MYB* into KU812, resulted in a significant decrease in MYB protein expression in KU812 cells (Figure 1D). Thus, based on these findings we postulated that MIR29B might activate *HBG* transcription, in part *via* binding the 3′-UTR of *MYB*.

**FIGURE 2:**
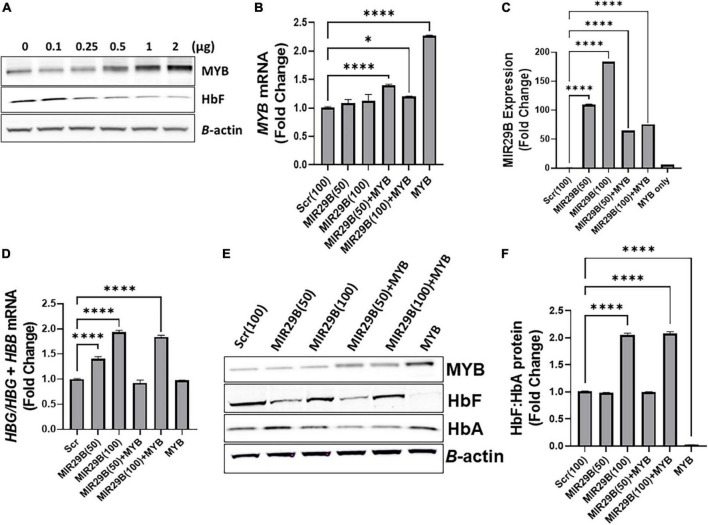
MicroRNA29B (MIR29B) mediates *HBG* reactivation *via* inhibiting *MYB* in KU812 cells. **(A)** Western blot analysis of MYB and HbF protein expression relative to β-actin protein in KU812 cells transfected with increasing concentrations of MYB plasmid DNA (μg). Fold change of **(B)** *MYB* mRNA relative to β-actin, **(C)** MIR29B expression relative to RNU48, and **(D)** *HBG*-to-*HBB* mRNA ratio as a function of *HBG*/*HBG* + *HBB* mRNA expression in KU812 cells following transfection for 48 h with MIR29B mimic (50 or 100 nm) alone or in combination with MYB overexpression plasmid vector (2 μg). **(E)** Western blot of MYB, HbF, and HbA protein expression with β-actin internal control. **(F)** Bar graphs showing quantification of the ratio of HbF-to-HbA protein in KU812 cells transfected with MIR29B alone or in combination with MYB. *P < 0.05 **P < 0.005, ***P < 0.0005, and ****P < 0.0001 is statistically significant.

To determine the extent to which MIR29B reactivates *HBG* gene expression *via MYB* gene silencing, we used a human tagged c-*MYB* ORF clone to overexpress *MYB* alone or when co-transfected with MIR29B mimic (50 or 100 nM) or Scr control (100 nM) into KU812 cells (Figure 2). Western blot analysis revealed that transfection of MYB plasmid DNA at a concentration of 2 μg resulted in the highest expression of MYB protein and lowest expression of HbF when compared to control cells (Figure 2A). Therefore, we used a concentration of 2 μg of MYB plasmid DNA to overexpress MYB in the remaining experiments. We further confirmed efficient transfection of *MYB* alone or when co-transfected with MIR29B (50 and 100 nM) into KU812 cells after 48 h by RT-PCR (Figure 2). Specifically, in KU812 cells co-transfected with MIR29B (50 and 100 nM) and *MYB*, we observed a significant increase in *MYB* mRNA expression as compared to Scr (100 nM) control cells and cells transfected with MIR29B alone (Figure 2B). Furthermore, we observed a drastic decrease, up to two-fold, in *MYB* mRNA expression in cells that were co-transfected with MIR29B (50 and 100 nM), which further suggests that MIR29B targets *MYB* resulting in its gene silencing.

Therefore, to determine whether MIR29B mediates *HBG* reactivation, in part *via MYB* gene silencing, we next quantified the ratio of *HBG*-to-*HBB* mRNA expression as a function of *HBG/HBG* + *HBB* using RT-qPCR analysis (Figure 2D). Based on our previous findings in KU812 cells, we observed a significant, up to a two-fold, increase in the ratio of *HBG*-to-*HBB* mRNA in cells transfected with MIR29B (50 and 100 nM) alone as compared to Scr control cells (*p* < 0.001). Furthermore, there was no significant difference in the ratio of *HBG*-to-*HBB* mRNA in KU812 cells transfected with *MYB* alone as compared to Scr control cells, which further confirms the role of MYB as an *HB*G repressor protein. Interestingly, in cells co-transfected with both MIR29B (100 nM) and *MYB*, the ratio of *HBG*-to-*HBB* mRNA yielded close to a two-fold increase which is similar to that of cells transfected with MIR29B alone (Figure 2D). In support of these findings, we further demonstrated a significant increase in HbF protein expression as a ratio of HbF-to-HbA in KU812 cells transfected with MIR29B (100 nM) alone or in combination with *MYB* as compared to Scr control cells (Figures 2E,F). We also confirmed the positive expression of MYB protein in cells transfected with MYB alone or in combination with MIR29B (50 and 100 nM). Flow cytometry analysis further confirmed the ability of MIR29B (50 and 100 nM) alone and in combination with MYB to increase the% of HbF-positive cells by up to 4.8-fold as compared to Scr control cells (Supplementary Figure 1). These findings further support our hypothesis that MIR29B mediates *HBG* reactivation and HbF induction in part *via MYB* gene silencing.

**FIGURE 3:**
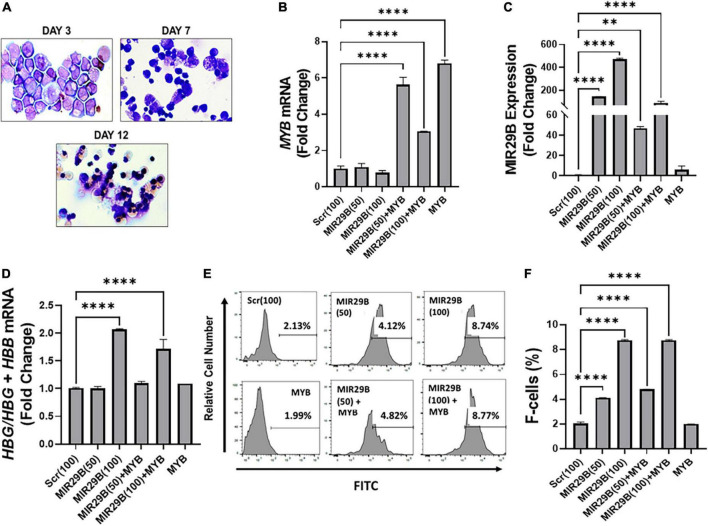
MicroRNA29B (MIR29B) mediates *HBG* reactivation *via MYB* gene silencing in normal human erythroid progenitors. **(A)** Wright-Giemsa-stained images of human erythroid progenitors (*n* = 3) undergoing differentiation at day 3, day 7, and day 12 *in vitro*. Quantitative RT-qPCR analysis of **(B)** *MYB* mRNA relative to β-actin, **(C)** MIR29B relative to RNU48, and **(D)** *HBG*-to-*HBB* mRNA ratio as a function of *HBG*/*HBG* + *HBB*, **(E)** Representative histograms from flow cytometry analysis of the percentage of HbF-positive cells following transfection of MIR29B alone or in combination with *MYB* into primary erythroid progenitors (*n* = 3) stained with FITC anti-HbF antibody. **(F)** Bar graphs showing quantification of the ratio of HbF-to-HbA in erythroid progenitors (*n* = 3) transfected with MIR29B alone or in combination with *MYB*. *P < 0.05, **P < 0.005, ***P < 0.0005, and ****P < 0.0001 is statistically significant.

We next performed studies under physiological conditions to further determine the extent to which MIR29B mediates *HBG* reactivation *via* inhibiting *MYB* using normal erythroid progenitors generated from human CD34^+^ stem cells in a liquid culture system as previously described (16, 21). Cell morphology examination by Giemsa staining confirmed erythroid lineage commitment by day 7 followed by the appearance of mature red blood cells by day 12 (Figure 3A). To compliment cell morphology, we also measured expression of the erythroid differentiation markers CD235a and CD71 by flow cytometry (Supplementary Figures 2A,B) to gain a better understanding of the effect of ectopic expression of MIR29B and *MYB* on erythroid maturation. The expression of CD71 did not significantly change following introduction of MIR29B alone; however, levels of CD71 decreased in cells that expressed *MYB* as compared to Scr control (Supplementary Figure 2A). Conversely, the percentage of CD235a positive cells significantly increased following MIR29B alone or in combination with *MYB* when compared to Scr control cells (Supplementary Figure 2B). Together, these data suggest that elevated levels of MIR29B and *MYB* may enhance erythroid maturation.

To further determine whether MIR29B and *MYB* were effectively delivered into the cells by electroporation, we measured MIR29B and *MYB* mRNA expression by RT-qPCR (Figures 3B,C). We observed a significant seven-fold increase in erythroid progenitors electroporated with *MYB* alone as compared to Scr control cells (Figure 3B). *MYB* mRNA expression was also confirmed in erythroid progenitors following co-electroporation with MIR29B (50 and 100 nM). Co-electroporation of erythroid progenitors with MIR29B (100 nM) and *MYB* significantly decreased *MYB* mRNA expression by 2.3-fold when compared to *MYB* alone (Figure 3B). In addition, we observed a dose-dependent increase up to 400-fold in MIR29B expression in cells transfected with MIR29B (50 and 100 nM) alone as compared to Scr control cells (*p* < 0.0001)(Figure 3C). This finding further supports the role of MIR29B in inhibiting *MYB* gene expression. This difference in expression of MIR29B between cells electroporated with MIR29B alone or co-electroporated with *MYB* is likely due to overexpression of *MYB*.

We further determined whether MIR29B mediates *HBG* activation *via* silencing *MYB* under physiological conditions. We co-expressed MIR29B (50 and 100 nM) and *MYB* into normal erythroid progenitors and measured the expression of *HBG*-to-*HBB* mRNA by RT-qPCR (Figure 3D). Real-time PCR analysis revealed a significantly higher *HBG*-to-*HBB* mRNA ratio by up to two-fold in erythroid progenitors electroporated with MIR29B (100 nM) alone or up to 1.5-fold when co-expressed with *MYB* as compared to Scr control cells (Figure 3D). Furthermore, there was no change in the ratio of *HBG*-to-*HBB* mRNA in erythroid progenitors electroporated with *MYB* alone when compared to Scr control cells (Figure 3D). In support of these findings, we further demonstrated by flow cytometry analysis the inhibitory effect of overexpressing MIR29B on MYB function (Figure 3E). Specifically, we confirmed a significant, up to four-fold, increase (*P* < 0.0001) in the percentage of HbF-positive cells among erythroid progenitors whether they overexpressed MIR29B alone (50 and 100 nM) or in combination with *MYB* (*P* < 0.0001) (Figures 3E,F). Furthermore, the percentage of HbF-positive cells in erythroid progenitors electroporated with *MYB* alone was similar to that of Scr control cells (Figures 3E,F). These studies support an important role for MIR29B in *MYB* and *HBG* gene regulation *in vitro*.

**FIGURE 4:**
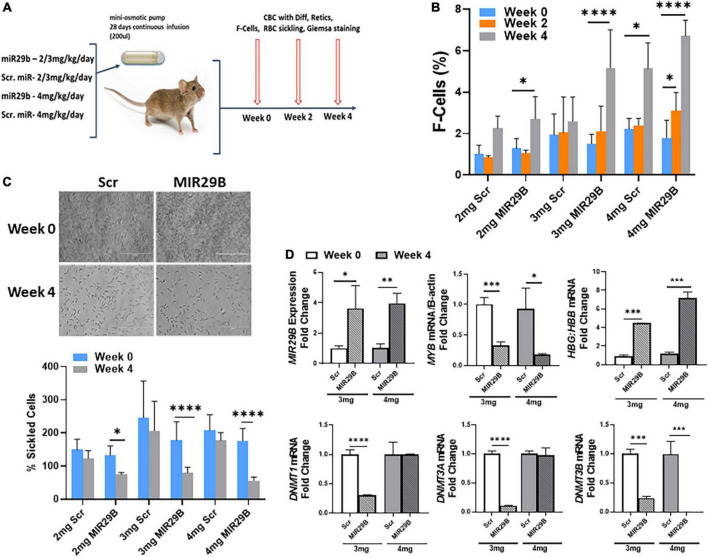
MicroRNA29B (MIR29B) induces HbF in humanized Townes sickle cell mice. **(A)** Townes SCD mice, which express human α-globin, γ-globin, and βS-globin genes and exhibit phenotypes including chronic hemolysis and sickling similar to human SCD, were surgically implanted with a mini-osmotic pump (Alzet) that continuously released MIR29B mimic at the concentration shown over 28 days. Age-and sex-matched mice (*n* = 4–10 per group) were treated and blood was collected at weeks 0, 2, and 4 for complete blood counts (CBC) with differential, reticulocytes, %F-cells, and red blood cell sickling under hypoxia conditions. Treatments consisted of 4 mice per group (2 males and 2 females) for MIR29B or SCR mimic at 2, 3, and 4 mg/kg/day and water vehicle and HU (100 mg/kg/day) controls. Tail–vein blood samples were collected in EDTA blood tubes at week 0, 2, and 4 along with weights. Evaluation of the effects of MIR29B on bone marrow hematopoiesis, were conducted by **(B)** Shown are bar graphs of the percentage of HbF-positive cells (F–cells) in erythroid progenitors isolated from Townes SCD mice and stained with fluorescein isothiocyanate-labeled anti-HbF antibody under the different treatment conditions. **(C)** Shown are bar graphs of the percentage of sickled cells obtained from freshly collected EDTA blood from sickle mice incubated under 1% hypoxia conditions for 12 h following the treatment conditions. **(D)** RT-qPCR analysis of MIR29B, *MYB*, *DNMT* gene expression and the *HBG*-to-*HBB* mRNA ratio as a function of *HBG*/*HBG* + *HBB* in mice treated with MIR29B or Scr control; *P < 0.05, **P < 0.005, ***P < 0.0005, and ****P < 0.0001 is statistically significant.

To advance the field and move novel small molecules from bench to bedside requires evidence of *in vivo* efficacy of HbF induction. Therefore, our final preclinical studies evaluated the potential of MIR29B to induce HbF using the humanized sickle cell mouse model. Townes sickle cell mice are an excellent model since they express human α-globin, γ-globin, and β^S^-globin genes and exhibit phenotypes including chronic hemolysis and sickling similar to human SCD. Mice 4–6 months old were administered MIR29B (2, 3, and 4 mg/kg/day) or Scr control (same concentration) to establish optimal dosing by continuous 4 weeks infusion using subcutaneous mini-osmotic pumps with six mice per treatment group (Figure 4A). At week 0, 2, and 4, mice were weighed and blood samples collected by tail bleed for automated CBC and reticulocyte percent, percentage of F-cells by flow cytometry. Over 4 weeks of treatment, no drug toxicity occurred or death (Supplementary Figure 3). Scr control mice had no significant change in blood counts over the 4 weeks treatment period, except for platelets at 3 mg/kg after 2 weeks (Supplementary Figure 3A). Treatment of mice with MIR29B (2–4 mg/kg/day) after 2 weeks caused a significant increase in the number of platelets 1.6-fold (*p* < 0.0005) and reticulocytes 1.7-fold (*p* < 0.05), suggesting that MIR29B stimulated erythropoiesis. By contrast, levels of total hemoglobin, hematocrit, and lymphocytes did not significantly change after MIR29B treatment (Supplementary Figures 3B,C). During MIR29B treatment, all mice exhibited normal behavior and had steady weight gain (Supplementary Figure 3D). At the end of treatment, pumps were removed to confirm that all medications were delivered.

We next analyzed the ability of MIR29B to induce HbF expression *in vivo*. As shown in Figure 4B, the percentage of F-cells significantly increased 2.1 and 3.4-fold in mice treated with 2 and 3 mg/kg MIR29B, respectively. We further demonstrated the anti-sickling effect mediated by MIR29B under hypoxic conditions (Figure 4C). Sickle erythroid precursors from Townes mice were incubated in 1% hypoxia conditions overnight, fixed with formaldehyde and examined by light microscopy. As shown in Figure 4D, MIR29B reduced the percentage of sickled erythroid precursors by up to 68% (*p* < 0.0005) after 4 weeks treatment, supporting the anti-sickling effects mediated by MIR29B. These findings support the ability of MIR29B to induce HbF *in vivo* in preclinical Townes sickle cell mice.

We previously showed that MIR29B functions as a DNA methyltransferase inhibitor by targeting *DNMT* gene expression *in vitro* (16). Since treatment of mice with 3 and 4 mg/kg MIR29B at 4 weeks significantly reduced the percentage of sickle cells, we measured mRNA expression from mice spleen tissue at week 0 and week 4 (Figure 4D). We confirmed a significant increase up to 3.8-fold in MIR29B expression in mice treated with 3 and 4 mg/kg MIR29B, respectively, compared to Scr control. Treatment of mice with 3 and 4 mg/kg MIR29B resulted in up to a 68% significant decrease in MYB mRNA expression (*p* < 0.005) and up to 76% for DNMT3B (Figure 4D). Similarly, treatment of mice with 3 mg/kg significantly decreased DNMT1 mRNA up to 70% and DNMT3A mRNA expression by 90%, while *HBG* mRNA shown as a function of *HBG*-to-*HBB* mRNA ratio significantly increased (Figure 4D), suggesting that MIR29B reactivated *HBG* gene expression in part *via* inhibiting *DNMT3* and *MYB* gene expression. Collectively, our findings support the ability of MIR29B to function as an HbF inducer for the treatment of β-hemoglobinopathies in part *via* targeting *MYB*.

## Discussion

MicroRNAs represent a novel class of small molecules that have gained much attention for their diagnostic and therapeutic potential to treat a wide variety of clinical diseases *via* targeting oncogenes and their gene products (22–25). MiRNAs are short (∼22 nucleotides) non-coding RNA molecules that associate with the miRNA-induced silencing complex (mRISC) and guide mRISC to silence specific mRNA in the cytoplasm (26). MicroRNAs facilitate mRNA degradation or suppression of translation by base-pairing to complementary sequences in the 3′-UTR of target mRNA (12, 26). We previously showed that MIR29B functions as an HbF inducer in KU812 cells and normal human erythroid progenitors by targeting *DNMT3* gene silencing (16). The discovery of novel small miRNA molecules, such as MIR29B, that target genes involved in *HBG* gene silencing to induce HbF levels will expand strategies to develop therapeutic options for SCD and other β-hemoglobinopathies.

Indeed, several miRNAs have been associated with HbF induction. In earlier studies by Bianchi et al. (27) they reported the upregulation of MIR210 in erythroid precursors of a thalassemia patient with high HbF levels. Additionally, they demonstrated that mithramycin targeted MIR210 as a mechanism of *HBG* activation in KU812 cells (27). In a subset of patients with SCD treated with hydroxyurea, expression of MIR26B, and MIR-151-3p was associated with HbF levels at maximum tolerated doses (28). Sangokoya et al. (29) were the first to report a correlation between MIR144 expression and the level of anemia in sickle cell patients and oxidative stress in sickle red blood cells due to reduced levels of the transcription factor NRF2. Following genome-wide miRNA expression profiling using reticulocytes isolated from SCD patients with extremes of HbF levels, our group confirmed increased MIR144 and reduced NRF2 levels in SCD patients with low HbF levels (21). Our findings provided additional supporting evidence for indirect mechanisms of HbF regulation by miRNAs (21). Previous studies by our group also demonstrated that MIR34A mediated HbF induction in KU812 cells by repression of STAT3 expression, another known repressor of *HBG* (30). Previously, Lee et al. (31) confirmed overexpression of LIN28B decreased miR-Let7 expression and increased HbF levels in primary erythroid cells. As further evidence of the therapeutic potential of miRNAs, MIR-486-3p induced HbF in adult erythroid progenitors by inhibiting BCL11A expression (32). MIR15A and MIR16-1 also induced HbF by targeting the *HBG* repressor MYB in infants with human trisomy 13 (33). Our recently published work demonstrated that MIR29B functions as an HbF inducer in KU812 cells and normal human erythroid progenitors by targeting *DNMT3* gene silencing (16). This is the first study to provide evidence of a miRNA that targets DNA methylation machinery as a mechanism of HbF induction. In that same study, we showed that introduction of MIR29B into KU812 cells further decreased MYB protein expression (16).

MYB is a well-characterized oncogene protein that also functions as an *HBG* repressor protein (34). In a genome-wide association study consisting of SCD and β-Thalassemia patients, the *HBS1L-MYB* intergenic region was associated with ∼17% of the inherited HbF variance in those patients (35). The transcriptional activator MYB is known to be essential for definitive hematopoiesis (36) and is highly expressed in immature hematopoietic cells and downregulated during erythropoiesis (37). Overexpression of MYB has been demonstrated to reduce HbF levels in KU812 cells (34). Knockdown of MYB in primary human erythroid progenitors has been reported to also induce HbF expression (33). Moreover, MYB regulates HbF expression in quantitative trait locus studies and functional assays (20, 28, 29, 33–35). MYB also indirectly silences *HBG* expression by activating KLF1 (Krüppel-like factor 1), a transcription factor that directly binds the *HBB* gene and BCL11A promoters during adult erythropoiesis (38).

Considering the function of MYB as an *HBG* repressor protein and our published study suggesting that MIR29B targets *MYB* in KU812 cells, we hypothesized that overexpression of MIR29B to silence *MYB* would mediate HbF induction. Using *in silico* analysis, we discovered that MIR29B has a consensus sequence complimentary to the 3′-UTR of *MYB*. We confirmed the ability of MIR29B to interact with the 3′-UTR of *MYB* using a luciferase reporter construct that expresses the *MYB* 3′-UTR. Introduction of MIR29B into KU812 cells and erythroid progenitors resulted in decreased MYB mRNA and protein, accompanied by an increase in HbF expression. Our findings support the ability of MIR29B to silence *MYB* through 3′UTR interactions. Collectively, our published studies support an essential role of MIR29B in modulating *HBG* transcription by two mechanisms involving DNA methylation and *MYB* gene silencing.

An excellent candidate for clinical development is MIR29B since it is a well-characterized tumor suppressor gene that functions as a DNMT inhibitor. To our knowledge, the only miRNA mimic that has previously undergone investigation in clinical trials was for liposomal injection of MIR34A mimic MRX43 for the treatment of advanced melanoma cancer (ClinicalTrials.gov Identifier: NCT01829971). Although this phase I clinical trial has now been terminated due to five immune related serious adverse events, the authors provided proof-of-concept for a miRNA-based therapy for cancer (39). MIR34A, similarly to MIR29B, regulates a broad number of genes involved in proliferation, metastasis, and chemo-resistance (40). Moreover, small RNA inhibitor molecules and antisense oligonucleotides, such as Patisiran (41) and Nusinersen (42), have been used in clinical trials for the treatments of hereditary transthyretin amyloidosis and infantile Spinal Muscular Atrophy, respectively. In the present study, we demonstrated for the first time the ability of MIR29B to increase HbF levels *via* inhibiting MYB in KU812 cells and normal human erythroid progenitors. We further showed the ability of MIR29B to induce HbF and reduce red blood cell sickling without producing adverse side effects in Townes sickle mice. Our findings further support the development of MIR29B as an HbF inducer in preclinical sickle transgenic mice.

## Data availability statement

The original contributions presented in the study are included in the article/supplementary materials, further inquiries can be directed to the corresponding author.

## Ethics statement

The animal study was reviewed and approved by the Augusta University Institutional Animal Care and Use Committee.

## Author contributions

AS-D designed the research study. QG, AS, EA-A, LL, UO, and BL performed the research. AS-D and BP contributed essential reagents and tools. AS-D, QG, AS, CP, and BL analyzed the data. AS-D and BP wrote several drafts of the manuscript. All authors reviewed the final draft of the manuscript.
