# Supplementary material for: MicroRNA29B induces fetal hemoglobin via inhibition of the HBG repressor protein MYB in vitro and in humanized sickle cell mice
Source: Front Med (Lausanne). 2022 Nov 25;9:1043686. doi: 10.3389/fmed.2022.1043686 (PMC9732025; doi:10.3389/fmed.2022.1043686)
Supplement: Supplementary file 1 [file Data_Sheet_1.docx]

MIR29B Induces Fetal Hemoglobin via Inhibition of the *HBG* Repressor Protein MYB in vitro and in humanized sickle cell mice

**Supplemental Material**

**Western blot analysis.** Total protein was isolated and Western blot analysis was performed as previously published (16). Primary antibodies against MYB (59995S), HbF (39386S), and HbA (84934S) were purchased from Cell Signaling Technology (Danvers, MA) and diluted in the range of 1:250 to 1:2000, incubated overnight and then followed by treatment with secondary antibody. The primary antibody against β-actin (AM4302), the internal control, was purchased from Invitrogen (Waltham, MA).

**Flow cytometry analysis.** To measure the percentage of HbF-positive cells (F-cells), erythroid progenitors were fixed with 1% formaldehyde and stained with Human HbF FITC conjugate antibody (ab19365, Abcam, Cambridge, UK). The isotype control IgG antibody (MBS524511, MyBioSource, San Diego, CA) was used to detect non-specific staining. Erythroid progenitor differentiation was determination by FITC-CD71 (555536) and FITC labeled CD235a (559943) antibodies staining (BD Biosciences, Franklin Lakes, NJ ). The percentage of HbF-positive cells levels and CD71 and CD235a expression were analyzed on a LSR-II flow cytometer (BD Biosciences, San Jose, CA) and the percentage of F‐cells was determined by FlowJo Version 10.0.7 software (Tree Star, OR) as previously reported (16, 19, 20).

| **Table I. Summary of primer sequences.** | |
| --- | --- |
| **RT-PCR primers 5’ 3’** | |
| *HBG* forward | GGCAACCTGTCCTCTGCCTC |
| *HBG* reverse | GAAATGGATTGCCAAAACGG |
| *HBB* forward | CTCATGGCAAGAAAGTGCTCG |
| *HBB* reverse | AATTCTTTGCCAAAGTGATGGG |
| *MYB* forward | AAGGGGACAGTCTGAATACCC |
| *MYB* reverse | AGGTTCCCAGGTACTGCTACA |
| β*-actin* forward | CTGGAACGGTGAAGGTGACA |
| β-*actin* reverse | AAGGGACTTCCTGTAACAACGCA |

**Supplementary Figure 1. MIR29B increases the percentage of HbF-positive cells in the presence of MYB.** Shown are flow cytometry histograms of KU812 cells under the different conditions after staining with FITC-conjugated anti-HbF antibody.

**
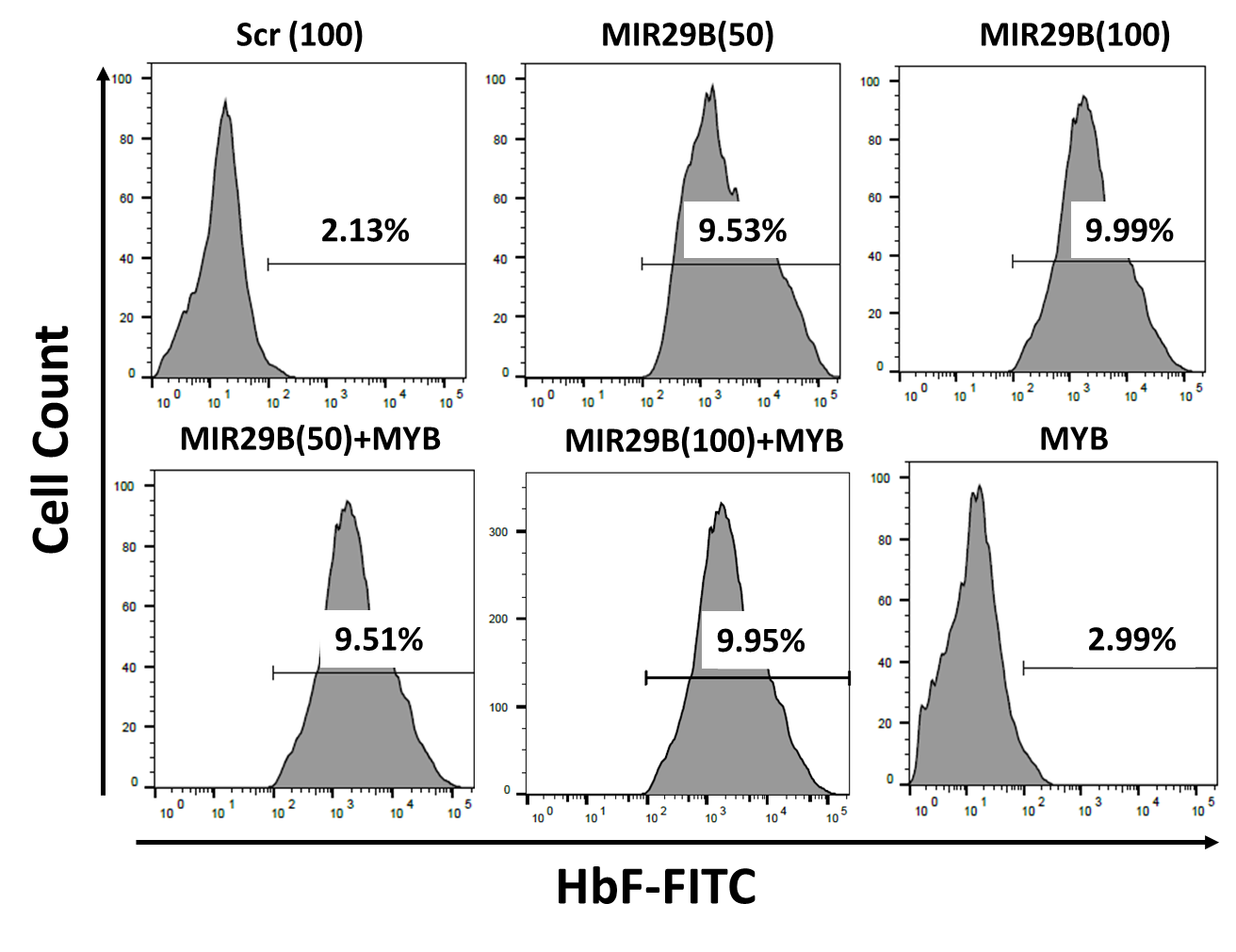
**

**Supplementary Figure 2. Erythroid marker expression during treatment with MIR29B and MYB.** Erythroid progenitors were electroporated with MIR29B (50 nM and 100 nM) or Scrambled control (100 nM) alone or in combination with MYB on day 6 and harvested on day 8. Expression of erythroid markers (A) CD235a and (B) CD71 were analyzed by flow cytometry. Data are shown at the mean + standard error of the mean.

**
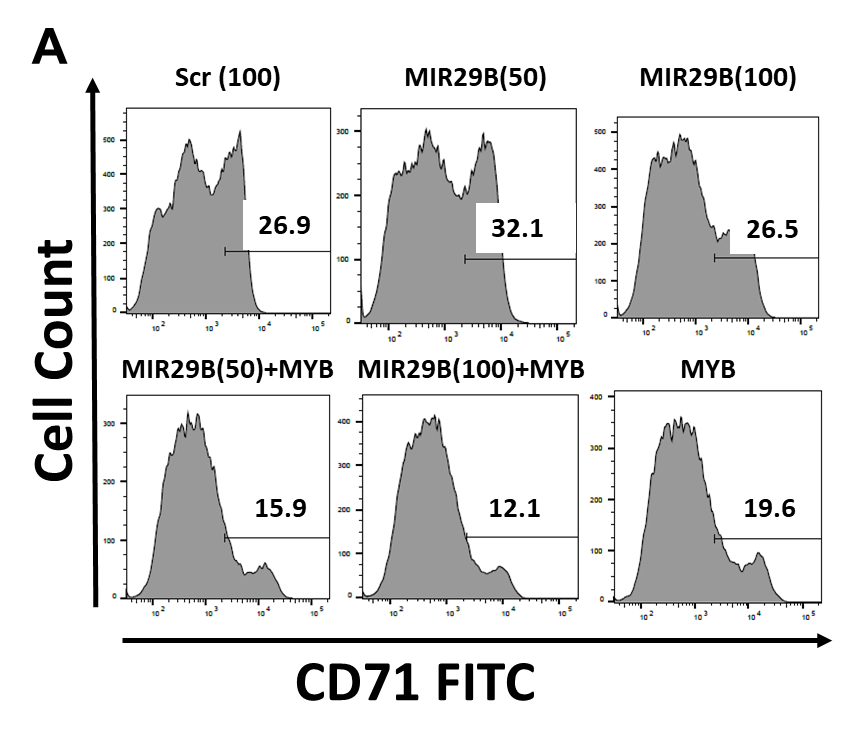
**

**
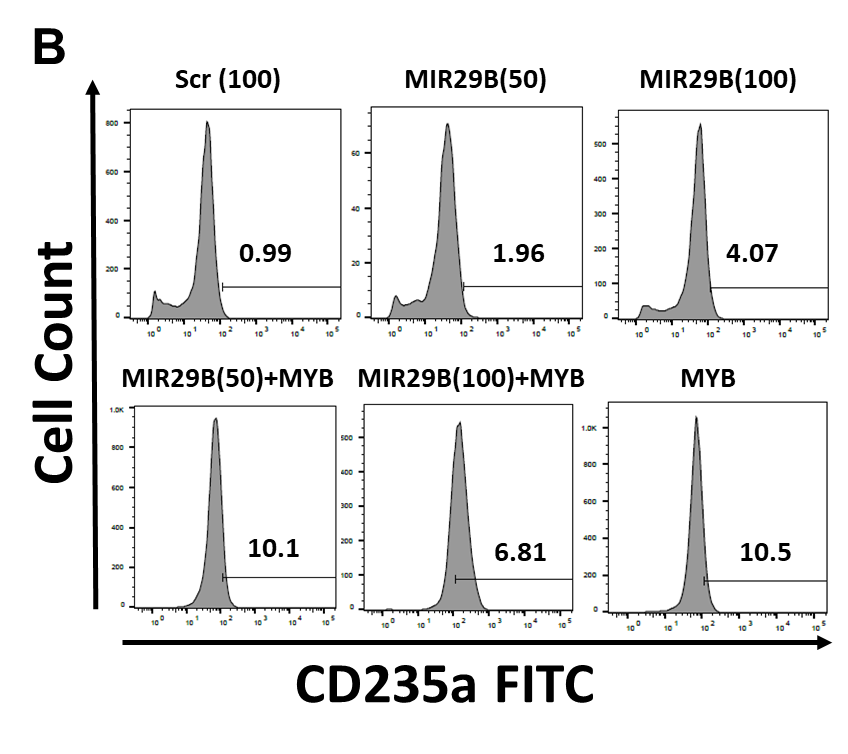
**

**Supplementary Figure 3. Complete blood counts with differential from Townes sickle mice treated with MIR29B or Scrambled (Scr) control.** Shown are bar graphs of (**A)** platelets, reticulocytes, red blood cells (rbc), and white blood cells (wbc), **(B)** hemoglobin and hematocrit, **(C)** lymphocytes and monocytes, and **(D)** weight in grams from blood of mice treated with different concentration of MIR29B or Scr at week 0 (blue), week 2 (orange) and week 4 (gray). **P<0.05*, ***P<0.005*, ****P<0.0005*, *****P<0.0001*

**
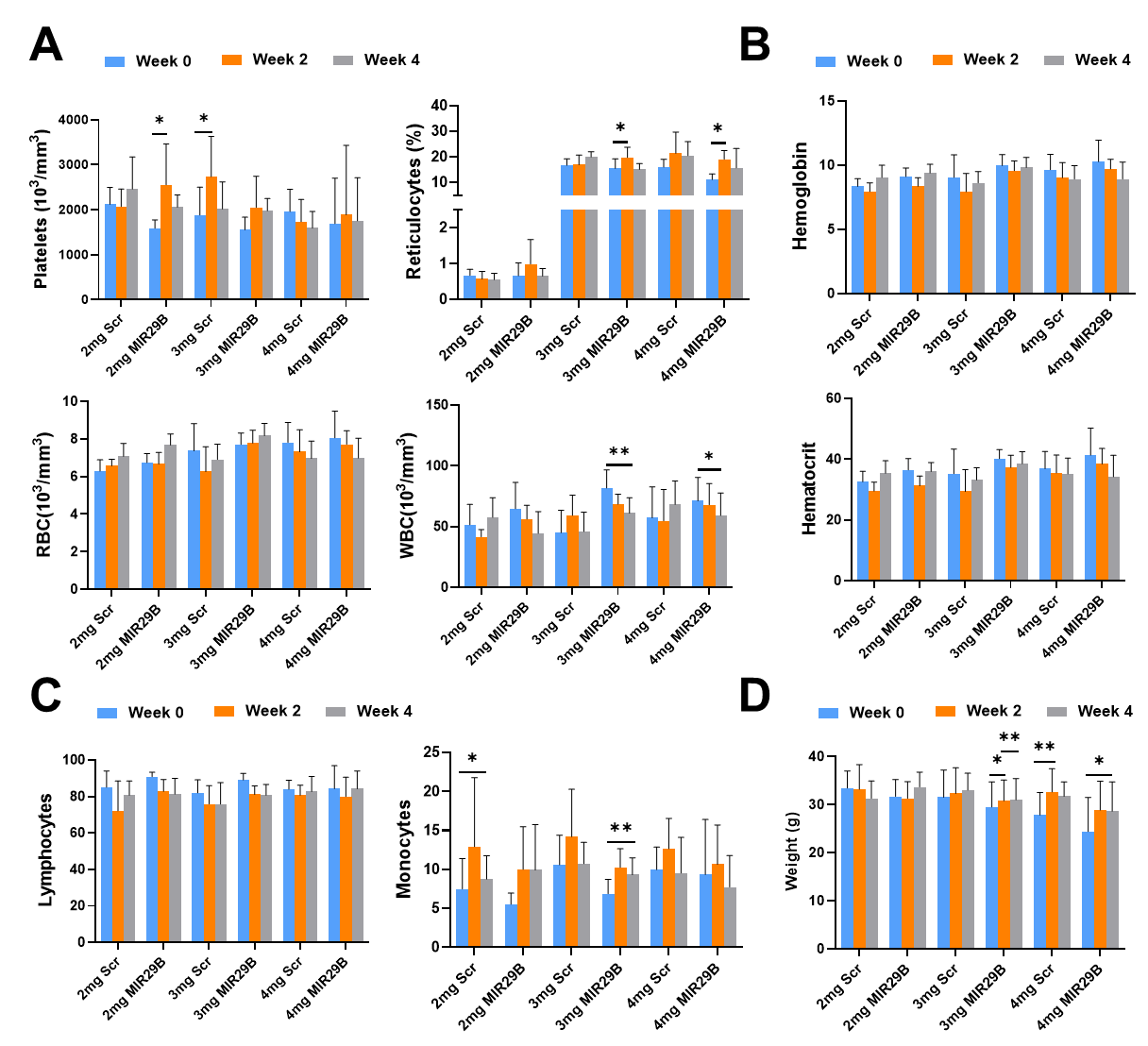
**
